# Applied Veterinary Informatics: Development of a Semantic and Domain-Specific Method to Construct a Canine Data Repository

**DOI:** 10.1038/s41598-019-55035-8

**Published:** 2019-12-09

**Authors:** Mary Regina Boland, Margret L. Casal, Marc S. Kraus, Anna R. Gelzer

**Affiliations:** 1 Department of Biostatistics, Epidemiology and Informatics, Perelman School of Medicine, University of Pennsylvania, Philadelphia, Pennsylvania, USA; 2 Institute for Biomedical Informatics, University of Pennsylvania, Philadelphia, Pennsylvania, USA; 3 Center for Excellence in Environmental Toxicology, University of Pennsylvania, Philadelphia, Pennsylvania, USA; 4 Department of Biomedical and Health Informatics, Children’s Hospital of Philadelphia, Philadelphia, Pennsylvania, USA; 5 Department of Clinical Studies and Advanced Medicine, School of Veterinary Medicine, University of Pennsylvania, Philadelphia, Pennsylvania, USA

**Keywords:** Experimental models of disease, Outcomes research, Translational research

## Abstract

Animals are used to study the pathogenesis of various human diseases, but typically as animal models with induced disease. However, companion animals develop disease spontaneously in a way that mirrors disease development in humans. The purpose of this study is to develop a semantic and domain-specific method to enable construction of a data repository from a veterinary hospital that would be useful for future studies. We developed a two-phase method that combines semantic and domain-specific approaches to construct a canine data repository of clinical data collected during routine care at the Matthew J Ryan Veterinary Hospital of the University of Pennsylvania (PennVet). Our framework consists of two phases: (1) a semantic data-cleaning phase and (2) a domain-specific data-cleaning phase. We validated our data repository using a gold standard of known breed predispositions for certain diseases (i.e., mitral valve disease, atrial fibrillation and osteosarcoma). Our two-phase method allowed us to maximize data retention (99.8% of data retained), while ensuring the quality of our result. Our final population contained 84,405 dogs treated between 2000 and 2017 from 194 distinct dog breeds. We observed the expected breed associations with mitral valve disease, atrial fibrillation, and osteosarcoma (P < 0.05) after adjusting for multiple comparisons. Precision ranged from 60.0 to 83.3 for the three diseases (avg. 74.2) and recall ranged from 31.6 to 83.3 (avg. 53.3). Our study describes a two-phase method to construct a clinical data repository using canine data obtained during routine clinical care at a veterinary hospital.

## Introduction

Understanding the origins of disease, including both environmental^1^ and genetic etiologies requires the use of good and validated models. Dogs are useful models for studying several canine and human diseases^2,3^, including cardiovascular diseases^4^, and various cancers^5^. Companion animals (sometimes called ‘pets’) are especially important because they develop disease spontaneously, which mirrors the process of disease progression in humans^6^. In addition, dogs and human share the same environmental exposures by living together. Hence if a disease is due in part to an environmental exposure, it is likely that both dogs and their humans would be exposed to the same environmental factor^4,7–9^.

In order to study disease prevalence and perform comparative analyses between humans and dogs, it is necessary to develop an accurate and validated data repository for the clinical data obtained during routine *veterinary* care at the Matthew J Ryan Veterinary Hospital of the University of Pennsylvania (PennVet). Informatics methods are required to develop and validate data repositories^10,11^. Research data repositories use data collected during routine clinical care. Unfortunately, data recorded during clinical care are often not collected for research purposes and therefore data entry errors occur frequently, and disease related terms can be used inconsistently^12^. Methods, including outlier detection, are often used in the human medical context to identify data anomalies and other issues with data collection^13^. Many of these techniques need to be tailored to the specific context. However, much prior work has been conducted in the human medical context with one study finding less then 2 articles published per year in the clinical veterinary informatics space^14^, therefore much information can be learned through application of these methods to a new domain. Some informatics work has been conducted in the veterinarian context^15^, but recent work has focused mainly in the Natural Language Processing domain^16,17^ and utilizing the UK/Australia VetCompass database^18^.

This study describes the development of a veterinary informatics method that enables the construction of a canine data repository validated using PennVet data, a veterinary hospital within the United States of America. This data repository could be used for cross-species comparisons between humans and canines to further our understanding of diseases that affect both species^4^.

## Materials and Methods

### Dataset

We obtained data obtained during routine clinical care for dogs treated at PennVet. These data are part of the PennVet Health Information System where patient histories are logged, hospital discharges are created, referral letters are generated and billing takes place. The PennVet Health Information System functions as an Electronic Medical Record (EMR) for veterinarians. To extract data for all dogs, electronically coded billing records were queried and records chosen with ‘canine’ in the species code field.

### Two-Phase framework for ensuring data quality

**Figure 1:**
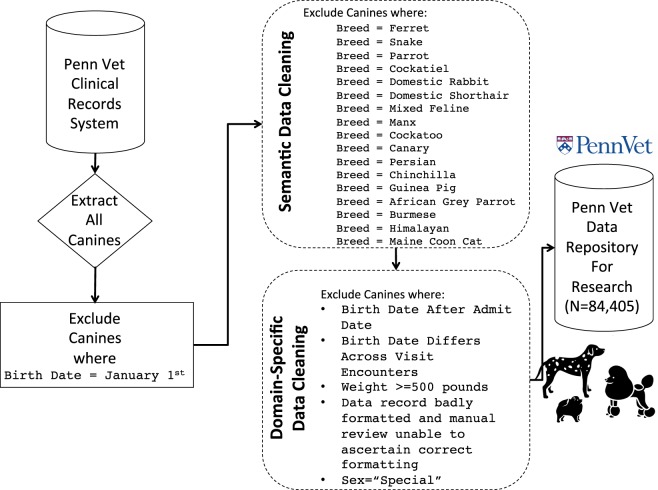
Schematic Diagram Illustrating the Construction of the Canine Data Repository at PennVet. All dog icons (“pomeranian”, “dalmatian”, “poodle”) within the figure are by: parkjisun, from thenounproject.com.

The veterinary context differs in several key ways from the traditional human medical system. For example, patient names (e.g., Fido) and birth dates are not required to be accurate for billing purposes. Therefore, January 1^st^ is often listed as the birth date if the true birth date is absent or unknown. In some instances, dogs are acquired from shelters or rescued and the exact date of birth is not known and therefore January 1^st^ is often entered as the birth date. Therefore, we removed all records where January 1^st^ was list (Fig. 1). We also excluded re-check visits so that each patient is counted once. Therefore, we only included initial diagnosis visits. We removed any duplicated records.

Our data cleaning^19^ algorithm was split into two phases: the first is a semantic data cleaning phase and the second consisted of domain-specific data cleaning phase. The semantic data cleaning phase involved removing all breeds that were not dog breeds. Data entry problems are endemic in human clinical records systems, and the veterinary context is no different. Therefore, during data entry in the clinic, a clinician or technician could set the animal field to canine accidently and then select a breed (e.g., ‘ferret’). Therefore, we excluded all breeds (e.g., ‘ferret’, ‘cockatiel’) that were not dogs. The breed name was selected from a drop-down list and therefore consisted of a controlled terminology (i.e., individuals could not add breed names in a free-text field). The controlled terminology consisted of a 4-letter breed code and a breed full name field. We manually reviewed the breed names to ensure that they were all dog breeds while excluding those that were not dog breeds. In addition, one dog breed named ‘Himalayan’ was deemed ambiguous because there is both a Himalayan cat breed and a Himalayan dog breed. Therefore we removed this ambiguous breed. Some breeds had more then 1 4-letter breed code to refer to them. For example the Miniature Dachshund was represented by breed codes MLHD and MDCH. We merged these two breed codes to ensure that each dog breed was represented by one unique code.

During the domain-specific data cleaning phase, we removed birth dates that occurred after the admit date, since animals are typically not admitted prior to their own birth. We also excluded patients where their birth date differs across visit encounters and where their weight was beyond 500 pounds as this signals a data entry error. To ensure that dog weights were logical (e.g., larger dog breeds having larger weights), we computed the average and standard deviations of weight across each dog breed and five distinct age categories. The five age categories were used because in addition to dog breed, age is also an important factor in dog weight. The 5 categories were ‘adolescent’ representing up to 1 year of age, ‘prime’ between 1 and 4, ‘second prime’ between 4 and 6, ‘elderly’ between 6 and 10 and ‘ancient’ being above 10.

**Figure 2:**
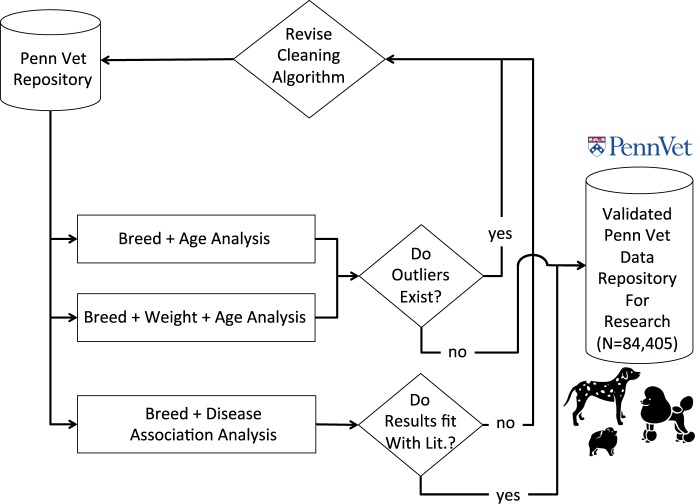
Schematic Diagram Illustrating the Validation and Iterative Refinement of Data Cleaning Method. If results of our age, breed and weight analysis or age and breed analysis revealed outliers, then we refined our data cleaning algorithm until we achieved results that were more inline with expected. In addition, if our disease – breed association analysis revealed peculiarities then we revisited our data cleaning algorithm until we achieved a cleaned and validated canine data repository. All dog icons (“pomeranian”, “dalmatian”, “poodle”) within the figure are by: parkjisun, from thenounproject.com.

We iteratively refined our algorithm for creating our research repository until we were satisfied that the data were adequately cleaned. The process for iteratively refining our algorithm is shown in Fig. 2. We investigated the relationship between dog breed and age, and also the relationship between dog breed, weight and age. If outliers were identified, then we revisited the cleaning process to make further changes. We also assessed each dog breed’s association with disease and compared with the literature. Disease breed association analyses and subsequent validation of those results are described in further details below.

### Case identification of mitral valve disease, atrial fibrillation and osteosarcoma

We identified each disease first by investigating the diagnostic codes. We explored the presence of disease-specific codes for each diagnosis type (e.g., primary or secondary diagnoses). For mitral valve disease, we used codes for ‘Acquired insufficiency mitral valve’ and ‘myxomatous mitral valve disease’; for atrial fibrillation, we used codes for ‘atrial fibrillation’ and for osteosarcoma, we used codes for osteosarcoma. There are codes for osteosarcoma that are specific to presence of the bone tumor in certain body locations. We ignored the location and focused on if osteosarcoma was diagnosed. Diagnoses were verified in the following way: mitral valve disease by echocardiography, atrial fibrillation by electrocardiogram (ECG), and osteosarcoma by radiographs plus/minus histopathology and/or cytology. The methods that veterinary clinicians perform to enter these diagnoses in the medical records is as follows: 1) the veterinary cardiologist who performed the echocardiography (gold standard to diagnose all structural heart disease such as valve disease and dilated cardiomyopathy) or acquired the electrocardiogram (gold standard to diagnose arrhythmias such as atrial fibrillation) identified all abnormalities and listed diagnoses such as mitral valve disease or atrial fibrillation in a drop down menu of disease names available in the EMR; Similarly for osteosarcoma, a veterinary radiologist, surgeon, oncologist or clinical pathologist made a diagnosis of osteosarcoma based on radiographs, cytology or a biopsy with a histological diagnosis of osteosarcoma and selected osteosarcoma from the dropdown menu of diagnoses. 2) These disease names all have corresponding medical codes in the EMR. The coding system does distinguish mitral valve disease due to congenital defects versus acquired mitral valve disease. However, the specific type of acquired disease is not delineated in the coding system (e.g., mitral valve disease due to dilated cardiomyopathy versus valve degeneration). For developing our research dataset, we exported dog records corresponding to each disease into an excel sheet for additional analyses.

One co-author (ARG) reviewed the medical records generated through this extraction process to make sure that the breeds and results in general made sense from a clinical perspective. The co-author (ARG) focused on validating the cases and not the control dogs. We did not have the resources to validate all 84,405 dogs in our dataset. However, this was part of our rationale for performing breed – disease association analyses to verify that our results were in general consistent with the literature (see later section). The breed-disease association results were also reviewed to ensure that they made sense from a clinical and canine genetic perspective.

We used only the first visit for each patient for both cases and controls. This first visit information is what we used for identifying age, weight and breed. However, when identifying whether a patient had a particular disease, for example, osteosarcoma, mitral valve disease or atrial fibrillation, all records were investigated for presence/absence of disease codes. Our resulting dataset only included 1 record per dog to facilitate statistical analysis.

### Information on breed identification in clinical veterinary records

At PennVet, breed identification is self-reported. Our work focuses on removing records with conflicting data (typically indicative of data entry errors or other issues). It is not feasible to review photos of dogs for our entire veterinary database and to ascertain if their owners’ assessment of the dog breed is accurate. Therefore, we are basing our assessment of the dog breed on the owner’s self-report. If a dog is a mixed breed of multiple ancestries it is listed as ‘mixed canine’. Our repository does not contain information on a dominant cross, unless explicitly specified (e.g., Goldendoodle). If a cross breed is explicitly named – such as ‘Goldendoodle’ it was not lumped into the mixed canine category, but was treated as its own breed even if it is not officially recognized by the American Kennel Club (AKC). The majority of dogs treated at PennVet are not AKC registered dogs, a major difference between our current work at PennVet and previous research involving registered dogs^7^. This is also reflected in that the majority of our dogs treated at PennVet are castrated, spayed or neutered (see Results).

### Association analyses to validate data accuracy

We chose to focus our evaluation on data quality^20^ rather than ease of use^21^ because we are interested in using this data repository for additional human-dog comparative research studies. Therefore, in addition to manual review to ensure data accuracy, we also performed several association analyses to validate our clinical data repository of 84,405 dogs. We validated our data repository by comparing results from our repository against the literature using several known breed–disease associations. We focused on mitral valve disease, atrial fibrillation, and osteosarcoma.

We performed fisher’s exact test to assess the significance of the relationship between each breed and the risk of either osteosarcoma or mitral valve disease. We then adjusted for multiple hypotheses using Bonferroni correction and also false discovery rate (FDR) correction using the Benjamini-Hochberg method. We used a gold-standard set of breeds associated with each disease (mitral valve disease, atrial fibrillation, and osteosarcoma) to compute the precision and recall. This ‘gold-standard’ set was derived from the literature on breeds associated with certain diseases^22^ and also breeds known to have a high incidence of the disease, regardless of whether an association test was explicitly performed. Precision is the number of findings that we reported as significant that agree with the literature while Recall is the number of findings from the literature that we successfully retrieve (or recapture in our results). We are especially focused on recall, which involves retrieving all the relevant findings reported in the literature.

### Empirical validation using permutation analysis

We used permutation analysis to compare our results with those obtained from the literature. For each disease, we developed a random cohort of patients that was the same size as the case population. For example, there were 717 mitral valve patients. Therefore, we set 717 random patients as having the disease. We then performed breed association analysis adjusting the p-values for multiple hypotheses using the FDR metric (less stringent), using 1000 random samples. We computed the precision and recall for each of these runs by comparing the findings from this random analysis with the gold standard.

## Results

### Two-Phase informatics framework for ensuring data quality

We started with a dataset containing 84,565 dogs. The first phase in our data quality framework was the semantic data cleaning phase. We selected all patient records where the species label was ‘canine’. However, several non-canine breeds were found indicating that the animal field was not correctly annotated. Therefore, we constructed an algorithm to exclude certain breed designations after manual review of the records. Our algorithm excluded the following non-canine breed designations: Ferret (N = 6), Snake (N = 1), Parrot (N = 1), Cockatiel (N = 2), Domestic Rabbit (N = 1), Domestic Shorthair (N = 74), Mixed Feline (N = 3), Manx (N = 1), Cockatoo (N = 1), Canary (N = 3), Persian (N = 2), Chinchilla (N = 2), Guinea Pig (N = 2), African Grey Parrot (N = 1), Burmese (N = 2), Himalayan (N = 2), Siamese (N = 2), and the Maine Coon cat (N = 3). This resulted in 109 records excluded. Note that removing breeds with the term ‘cat’ in their title would be an insufficient data cleaning approach as certain canine breeds, including the ‘Australian Cattle’ Dog and the ‘Catahoula Leopard’ Dog contain the word ‘cat’ in their names. Therefore, we manually reviewed all excluded breeds to ensure that they were in fact not legitimate dog breeds.

During the domain-specific data cleaning phase, we removed patient records (N = 5) where the birth date occurs after the admit date since animals are typically not admitted prior to their own birth and this likely represents a coding error. For patient sex, one of five standard concepts were recorded. These include F: Female, M: Male, H: Hermaphrodite, S: Special, U: Unknown. We excluded patients where a random numeric character was used for patient sex and it was not clear how to convert this to the standardized sex characterization (N = 2). In addition, we excluded patients that were listed with ‘Special’ as their sex (N = 18). The ‘Special’ sex was recorded for various peculiar instances. For example, where a whole litter of puppies were inoculated or spayed/neutered at the same time and hence the animal id was not for an individual patient, but a group. Therefore, we chose to exclude those of ‘Special’ sex. We also excluded patients where their birth date differs across visit encounters (N = 1). We also excluded patient records where the weight was beyond 500 pounds (N = 19). We calculated the average dog weight per breed and age category (described in Materials and Methods). The averages and standard deviations for each dog breed are given in Supplemental File 1. We also provide the average age and standard deviation for each dog breed, given in Supplemental File 2. We also excluded 1 record with a null animal id (N = 1). We selected only the first diagnosis visit per visit (described in methods), but there was still one patient with multiple visits in the dataset. Therefore, we chose their first visit and excluded the second later visit to make our dataset consistent throughout. Our initial query excluded dogs with a January 1^st^ birth date. However, we performed an additional check and found 5 dog records that were still included in our sample, therefore we excluded these at this stage (N = 5). However the true number of dogs with a January 1^st^ birth date was not assessed for the entire database as a whole and is much larger then this small set of 5 dogs. Our final dataset consisted of 84,405 unique dogs. In total, 160 dogs were removed or 0.2% of the original dataset. Therefore, our method retains a large portion of the data (99.8% retained).

### Dataset

**Figure 3:**
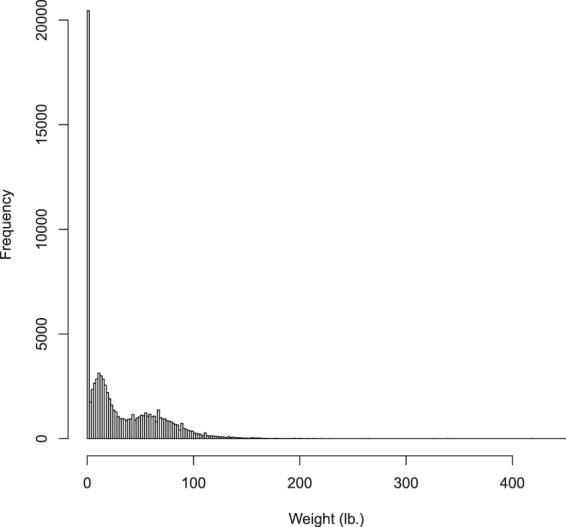
Histogram of Weight Across All Dog Breeds and Ages in Our PennVet Canine Data Repository. Note that weight was missing for data obtained in year 2012 and 2013 and therefore it was set to 0. This accounts for the large spike at 0 in Fig. 3.

**Figure 4:**
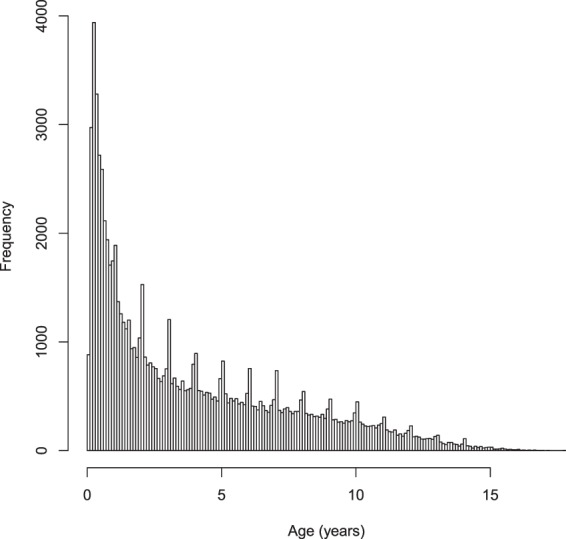
Average Age Across All Dog Breeds in Our PennVet Canine Data Repository.

Our final dataset contains 84,405 dogs treated at PennVet between 2000 through 2017. These dogs come from 194 distinct breeds (where mixed breed is considered as a unique breed). Table 1 contains the prevalence of each breed in our dataset for the 25 most common breeds. The most common breed was ‘mixed breed’, followed by Labrador Retrievers, American Pitbull Terrier, Golden Retriever, German Shepherd, and Yorkshire Terrier. The least common breeds were the Otterhound, Harrier and the Caucasian Ovcharka (sometimes called the Caucasian Shepherd Dog). For patient sex, our final dataset contained 45,255 males, 38,904 females, 240 unknown, and 6 hermaphrodites. The average weight across all dogs in our dataset is given in Fig. 3. Note that weight was missing for data obtained in year 2012 and 2013 and therefore it was set to 0. The average age across all dogs in our dataset is given in Fig. 4. We found that 24,651 dogs were spayed, 27,198 were castrated and 419 were reported as being intact (i.e., not spayed or neutered). We also found that 31,403 dogs had missing neutered status and 734 were reported as ‘unidentified’ with regards to their spayed/neutered status.

**Table 1:** Twenty-five Most Common Breeds at PennVet.

Breed Name	No. Seen at PennVet
Mixed Canine	21087
Labrador Retriever	5215
American Pit Bull Terrier	4241
Golden Retriever	2987
German Shepherd	2693
Yorkshire Terrier	2687
Chihuahua	2341
Shih Tzu	2132
Boxer	2128
English Bulldog	1967
Unspecified	1632
Dachshund	1610
Rottweiler	1531
Pug	1456
Pomeranian	1194
Beagle	1143
Maltese	1121
Jack Russell Terrier	1003
Bichon Frise	985
Cocker Spaniel	932
Cavalier King Charles Spaniel	930
Boston Terrier	896
Great Dane	724
French Bulldog	704
Doberman Pinscher	701

### Comparison of association analyses from pennvet with literature to validate data accuracy

For data validation purposes, we compared the results from our datasets to those in the literature for disease – breed associations. We investigate the association between certain dog breeds and three specific diseases – mitral valve disease, atrial fibrillation and osteosarcoma. We focus on these three diseases as breed proclivities are published^22^.

#### Mitral valve disease

Using our dataset of cleaned PennVet data, we investigated associations between certain dog breeds and risk of mitral valve disease. We report results both for those that are significant after correcting for multiple hypotheses using Bonferroni and false discovery rate (FDR) correction using Benjamini-Hochberg. Results are shown in Table 2. A total of 717 cases of mitral valve disease were diagnosed between 2000 and 2017 at PennVet. We found the following breeds were predisposed to mitral valve disease after adjusting for multiple testing using the Bonferroni correction method (most robust method): Cavalier King Charles Spaniel (Odds Ratio or OR = 14.55, 95% CI: 11.44, 18.34), Norfolk Terrier (OR = 55.87, CI: 22.22, 130.26), Maltese (OR = 3.07, CI: 2.02, 4.50), Whippet (OR = 15.56, CI: 5.95, 34.49), Chihuahua (OR = 2.15, CI: 1.52, 2.95), and the Airedale Terrier (OR = 6.83, CI: 2.88, 13.91). Many of these findings are concordant with the literature, including the association between Cavalier King Charles Spaniels and increased risk of mitral valve disease^23^. In addition, we found the following breeds were protected against mitral valve disease, including the American Pit Bull Terrier (OR = 0.03, CI: 0.00, 0.15), Mixed breeds (OR = 0.51, CI: 0.41, 0.63), German Shepherds (OR = 0.38, CI: 0.17, 0.73) and Labrador Retrievers (OR = 0.17, CI: 0.07, 0.34).

**Table 2:** Breed Associations for Mitral Valve Disease – Concordance with Literature.

Breed	No. in Penn Vet	No. with Mitral Valve Disease (N = 717)	Odds Ratio (OR)	OR 95% CI	Adjusted P-value FWER*	Adjusted P-value FDR**	Concordant with Literature	Ref.
Cavalier King Charles Spaniel	930	92	14.55	11.44, 18.34	2.7 × 10^−65^	2.7 × 10^−65^	Yes	^24^
**American Pit Bull Terrier**	**4241**	**1**	**0**.**03**	<**0**.**001**, **0**.**15**	**1**.**1** × **10**^**−12**^	**5**.**3 × 10**^**−13**^	**Novel?**	
Norfolk Terrier	28	9	55.87	22.22, 130.26	2.5 × 10^−10^	8.5 × 10^−11^	Yes	^22^
**Mixed Canine**	**21087**	**104**	**0**.**51**	**0**.**41**, **0**.**63**	**1**.**5 × 10**^**−9**^	**3**.**7 × 10**^**−10**^	**Novel?**	
**Labrador Retriever**	**5215**	**8**	**0**.**17**	**0**.**07**, **0**.**34**	**3**.**0 × 10**^**−9**^	**5**.**9 × 10**^**−10**^	**Yes**	^24^
Maltese	1121	28	3.07	2.02, 4.50	1.2 × 10^−4^	2.0 × 10^−5^	Yes	^25,26^
Whippet	60	7	15.56	5.95, 34.49	1.6 × 10^−4^	2.3 × 10^−5^	Yes	^24^
Chihuahua	2341	41	2.15	1.52, 2.95	3.4 × 10^−3^	4.2 × 10^−4^	Yes	^24^
Airedale Terrier	146	8	6.83	2.88, 13.91	7.6 × 10^−3^	8.5 × 10^−4^	^§^Related	
Great Dane	724	16	2.67	1.51, 4.40	0.11	0.01	Yes	^22^
**German Shepherd**	**2693**	**9**	**0**.**38**	**0**.**17**, **0**.**73**	**0**.**25**	**0**.**02**	**Yes**	^24^
Bull Terrier	106	5	5.81	1.84, 14.08	0.42	0.04	Yes	^22^
Doberman Pinscher	701	14	2.41	1.30, 4.09	0.60	0.05***	§Related	
**Pug**	**1456**	**3**	**0**.**24**	**0**.**05**, **0**.**70**	**0**.**67**	**0**.05***	**Novel?**	

*Adjusted for the Family-Wise Error Rate using Bonferroni Correction Method.

**Adjusted for False Discovery Rate using Benjamini-Hochberg Method.

***P-values round up to 0.05, but are actually less then 0.05.

^§^Related: Breed associated with cardiomyopathy^22^.

Rows highlighted in bold represent breeds that are protected against mitral valve disease (OR < 1).

In the literature^22^ the following breeds were at increased risk of mitral valve disease: Great Danes, Beagles, Golden Retrievers, Bull Terrier, Norfolk Terrier and Dachshunds. A large-population study using the VetCompass data from the United Kingdom^24^ found that the following breeds were at risk for mitral valve disease: the Cavalier King Charles Spaniel^24^, King Charles Spaniel^24^ (in our dataset this breed was considered the same as the Cavalier King Charles Spaniel), Chihuahua^24^, Whippet^24^, Shih Tzu^24^, Yorkshire Terriers^24^, Border Collies^24^, Miniature Schnauzer^24^, and Poodles^24^ (in our dataset, we had three different sizes of Poodles)^24^. They also found that Labrador Retrievers^24^ and German Shepherds^24^ were protected against mitral valve disease^24^, findings confirmed in our dataset (Table 2) along with the Staffordshire Bull Terriers^24^. Another study found Cavalier King Charles Spaniel^25,26^, Chihuahua^25,26^, Maltese^25,26^, Pekingese^25,26^, and only toy and miniature Poodles (not standard Poodles) as predisposed^25,26^ to mitral valve disease.

Nine of the fourteen breeds associated with mitral valve disease in our study were reported in the literature as being significantly associated with mitral valve disease (either protected or at risk breeds) (Table 2**)**. This resulted in a precision of 64.3% and recall of 45.0% (Table 3). There were eleven breeds reported in the literature as being associated with mitral valve disease that were not found associated in our study, these include: Beagles, Standard Poodle, Toy Poodle, Miniature Poodle, Golden Retrievers, Dachshunds, Miniature Schnauzer, Pekingese, Shih Tzu, Yorkshire Terrier and Staffordshire Bull Terrier (protected against mitral valve disease). This resulted in a low recall of 45.0% (Table 3). As stated above, one study found that only toy and miniature Poodles were associated with mitral valve disease^25,26^, and another found poodles in general^24^. Our dataset contains all three different sized poodles as distinct breeds, and we found none to be associated with mitral valve disease. Two breeds associated with mitral valve disease in our dataset are known to be at risk for cardiomyopathy, including the Airedale Terrier^27^ and the Doberman Pinscher. We found the American Pit Bull Terrier was protected against mitral valve disease (Table 2), which was not reported in the literature previously.

**Table 3:** Precision and Recall for Algorithm Using Breed – Disease Associations.

Disease	No. Breeds Associated in Literature seen at PennVet	Precision	Precision P-value*	Recall	Recall P-value*
Mitral Valve Disease	20	9/14 (64.29%)	0.004	9/20 (45.00%)	<0.001
Atrial Fibrillation	6	5/6 (83.33%)	<0.001	5/6 (83.33%)	<0.001
Osteosarcoma	19	6/10 (60.00%)	0.037	6/19 (31.58%)	<0.001
Osteosarcoma (+only)**	19	6/8 (75.00%)	0.006	6/19 (31.58%)	<0.001
Overall Mean Across All 3 Diseases***		74.21%		53.30%	

*P-values determined by comparison with 1,000 random permutations.

**Only Looking at Positive Findings to Calculate Precision Because All Published Research is on Associated Breeds and Does Not Investigate Breeds Protected Against Osteosarcoma.

***Mean Uses the Osteosarcoma Positive Only (because no negative results reported in literature).

#### Atrial fibrillation

Likewise, using our PennVet dataset, we investigated the association between breed and risk of atrial fibrillation. Overall, 127 dogs admitted between 2000 and 2017 were diagnosed with atrial fibrillation. We found that five breeds were at increased risk of atrial fibrillation using Bonferroni while six were found to be at increased risk when using the FDR p-value correction method (Table 4). We found no breeds that were protected against developing atrial fibrillation. The breeds at risk for atrial fibrillation include: Great Dane (OR = 14.58, 95% CI: 7.69, 25.65), Newfoundland (OR = 17.41, CI: 7.29, 35.84), Neapolitan Mastiff (OR = 34.20, CI: 8.96, 93.35), Doberman Pinscher (OR = 7.02, CI: 2.76, 15.00) and the Irish Wolfhound (OR = 29.10, CI: 5.78, 90.60). The Mastiff (OR = 6.48, CI: 2.06, 15.63) was significantly associated with risk of atrial fibrillation if the FDR p-value adjustment method was used (Table 4). The breeds that are reported to have the highest risk of atrial fibrillation in the literature are: Irish wolfhounds, Great Danes^22^, Newfoundland and Doberman Pinschers^28–32^ along with Mastiff (unspecified type)^22^ and Rottweilers^22^. Therefore, we achieved a precision of 83.3% (5/6) and recall of 83.3% (5/6).

**Table 4:** Breed Associations for Atrial Fibrillation – Concordance with Literature.

Breed	No. in Penn Vet	No. with Atrial Fibrillation (N = 127)	Odds Ratio (OR)	OR 95% CI	Adjusted P-value FWER*	Adjusted P-value FDR**	Concordant with Literature	Ref.
Great Dane	724	14	14.58	7.69, 25.65	1.3 × 10^−9^	1.3 × 10^−9^	Yes	^22,28–32^
Newfoundland	332	8	17.41	7.29, 35.84	9.1 × 10^−6^	4.6 × 10^−6^	Yes	^28–32^
Neapolitan Mastiff	84	4	34.20	8.96, 93.35	1.7 × 10^−3^	5.5 × 10^−4^	^**§**^**Related**	
Doberman Pinscher	701	7	7.02	2.76, 15.00	0.02	4.8 × 10^−3^	Yes	^22,28–32^
Irish Wolfhound	73	3	29.10	5.78, 90.60	0.04	7.4 × 10^−3^	Yes	^28–32^
Mastiff	535	5	6.48	2.06, 15.63	0.26	0.04	Yes	^22^

*Adjusted for the Family-Wise Error Rate using Bonferroni Correction Method.

**Adjusted for False Discovery Rate using Benjamini-Hochberg Method.

^§^Related: Breed associated with cardiomyopathy^22^.

#### Osteosarcoma

We also chose one non-cardiovascular disease to validate the PennVet database against, namely Osteosarcoma. Overall, 307 dogs admitted between 2000 and 2017 were diagnosed with Osteosarcoma and treated at PennVet. We found 8 breeds were at increased risk of Osteosarcoma using the Bonferroni method including, the Rottweiler (OR = 7.34, 95% CI: 5.01, 10.46), Greyhound (OR = 14.80, CI: 8.81, 23.61), Labrador Retriever (OR = 2.42, CI: 1.70, 3.36), St. Bernard (OR = 11.39, CI: 4.47, 24.26), Great Dane (OR = 5.19, CI: 2.72, 9.06), Irish Wolfhound (OR = 11.84, CI: 2.37, 36.36), and Bullmastiff (OR = 10.90, CI: 4.60, 22.15) (Table 5). One breed was protected against developing Osteosarcoma, namely the Yorkshire Terrier (OR = 0.00, CI: 0, 0.37) (Table 5). Using the less stringent FDR p-value correction method revealed that one more breed was at increased risk of Osteosarcoma namely the Anatolian Shepherd Dog (OR = 45.90, CI: 4.97, 208.26) while another breed was protected against Osteosarcoma, namely the Shih Tzu (OR = 0.00, CI: 0, 0.47) (Table 5**)**.

**Table 5:** Breed Associations for Osteosarcoma – Concordance with Literature.

Breed	No. in Penn Vet	No. with Osteosarcoma (N = 307)	Odds Ratio (OR)	OR 95% CI	Adjusted P-value FWER*	Adjusted P-value FDR**	Concordant with Literature	Ref.
Rottweiler	1531	36	7.34	5.01, 10.46	2.6 × 10^−16^	2.6 × 10^−16^	Yes	^22,33,34^
Greyhound	414	20	14.80	8.81, 23.61	2.7 × 10^−14^	1.3 × 10^−14^	Yes	^33^
Bullmastiff	214	8	10.90	4.60, 22.15	2.7 × 10^−4^	9.0 × 10^−5^	Novel?	
Labrador Retriever	5215	42	2.42	1.70, 3.36	3.8 × 10^−4^	9.6 × 10^−5^	Yes	^22,35^
Great Dane	724	13	5.19	2.72, 9.06	6.4 × 10^−4^	1.3 × 10^−4^	Yes	^22^
St. Bernard	179	7	11.38	4.47, 24.26	9.3 × 10^−4^	1.5 × 10^−4^	Yes	^34^
**Yorkshire Terrier**	**2687**	**0**	**0**.**000**	**0**, **0**.**37**	**0**.**02**	**3**.**0 × 10**^**−3**^	**Novel?**	
**Shih Tzu**	**2132**	**0**	**0**.**000**	**0**, **0**.**47**	**0**.**14**	**0**.**02**	**Novel?**	
Anatolian Shepherd Dog	14	2	45.90	4.97, 208.26	0.23	0.03	Novel?	
Irish Wolfhound	73	3	11.84	2.37, 36.36	0.48	0.05***	Yes	^34^

*Adjusted for the Family-Wise Error Rate using Bonferroni Correction Method.

**Adjusted for False Discovery Rate using Benjamini-Hochberg Method.

***P-values round up to 0.05, but are actually less then 0.05.

Rows highlighted in bold represent breeds that are protected against osteosarcoma (OR < 1).

Twenty breeds are predisposed to Osteosarcoma in the literature including, Rottweiler^22,33,34^, Labrador Retriever^22,35^, Golden Retriever^34^, Flat-coated retriever^34^, German Shepherd^36^, Greyhound^33^, Doberman Pinscher^34^, Boxer^22,34^, Great Dane^22,33,34^, Saint Bernard^34^, Mastiff^22,35^, Great Pyrenees^37^, Newfoundland^22,34^, Hovawart (not treated at PennVet)^34^, Bernese Mountain Dog^34^, Leonberger^34^, Standard Schnauzer^34^, Irish Setter^34,38^, and Irish Wolfhound^34^. Collies were also reported as a high incidence breed, but not having a high relative risk of disease^39–41^.

We failed to find the Golden Retriever, German Shepherd, Doberman Pinscher, Mastiff, Great Pyrenees, Leonberger, Boxer, Flat-coated Retriever, Hovawart (not treated at PennVet), Standard Schnauzer, Irish Setter, Collie, and Bernese Swiss Mountain Dog breeds at increased risk for Osteosarcoma. Assuming that the literature is 100% accurate, then the recall for Osteosarcoma is (6/19) or 31.6% (Table 3). The most common dog breeds predisposed to Osteosarcoma as reported in the literature are Greyhound, Rottweiler and the Great Dane^33^. We found all three of these breeds to be predisposed to Osteosarcoma in the cleaned PennVet dataset (Table 5).

#### Precision and recall

Precision measures the number of retrieved breed – association results by our algorithm versus those reported in the literature. Recall measures how many of the literature associations we can replicate in our study (Table 3). This precision/recall analysis assumes that we are equally powered to detect all breed – disease associations (which may not be the case for some rarer breeds) and also that the reported associations in the literature are true. For the purposes of this study, recall is more important as not all breeds predisposed to certain diseases are known (again this comes down to a power issue). For mitral valve disease both dog breeds at risk for and protected against the disease have been reported^24^ therefore, computing precision and recall on the entire set was logical. However, for osteosarcoma, only at-risk breeds were reported in the literature. Therefore, we computed precision and recall for both at risk breeds only (positive or + only in Table 3) and the full set of at-risk and protected breeds (Table 5). Including the breeds protected against osteosarcoma reduces the precision as no studies in the literature reported breeds protected against osteosarcoma (precision = 60.0% vs. 75.0%, Table 3). Overall three diseases, precision ranged from 60.0% to 83.3% and recall ranged from 31.6% to 83.3% depending on the disease of interest (Table 3).

#### Empirical validation

We also compared our precision and recall results with those obtained from our permutation analysis. We randomly generated a ‘diseased’ cohort that was the same size as our comparison disease cohort (e.g., 717 patients for the mitral valve disease, 127 for the atrial fibrillation disease, 307 for osteosarcoma). Therefore, each random ‘diseased’ cohort was specific to the disease. We then performed breed association analysis adjusting the p-values for multiple hypotheses using the FDR metric (less stringent). We computed the precision and recall by comparing the findings from this random analysis with the gold standard, using 1,000 random samples per disease.

**Figure 5:**
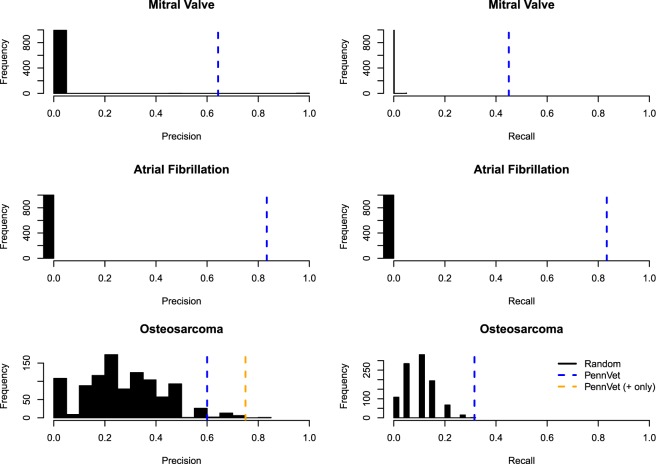
Precision and Recall for PennVet vs. Random for Three Diseases in Our Test Set: Mitral Valve Disease, Atrial Fibrillation and Osteosarcoma. We found higher precision and recall for all three diseases in test set: Mitral Valve Disease, Atrial Fibrillation and Osteosarcoma in our cleaned PennVet dataset versus the random set. For each disease, we developed a random cohort of patients that was the same size as the case population. For example, there were 717 mitral valve patients. Therefore, we set 717 random patients as having the disease. We then performed breed association analysis adjusting the p-values for multiple hypotheses using the FDR metric. This was performed 1000 times for each disease. Distributions of the precision and recall are shown above in Fig. 5.

We found that our PennVet dataset significantly outperformed the random samples for both precision and recall for all three diseases (P < 0.05, Fig. 5). Because the gold-standard set of breeds for Atrial Fibrillation was small (6 breeds associated in the literature), the precision and recall for the random samples approached 0. On the other hand, breeds at risk for osteosarcoma are very common, including four of the top ten breeds treated at PennVet (Table 1), namely Labrador Retrievers, Golden Retrievers, German Shepherds and Boxers. However, among these common breeds, we only found 3/4 to be at risk of Osteosarcoma (Table 5), and failed to find Golden Retrievers to be at risk of Osteosarcoma. Therefore, random spurious correlations between various breeds and risk of osteosarcoma were more likely Fig. 5. Importantly, our cleaned PennVet dataset significantly outperformed random for all three diseases, including osteosarcoma (P < 0.05, Table 3, Fig. 5**)**. Furthermore, if we only investigate positive associations the precision for osteosarcoma was high (75.0%, p = 0.003).

## Discussion

Overall, our study demonstrates that a clinical data repository can be constructed using dog data obtained during routine clinical care at a veterinary hospital (PennVet). Our two-step framework allowed us to maximize data retention (99.8% of data retained). We assessed the data quality by performing high-throughput breed association analyses for three key diseases and compared our findings with those in the literature. Our cleaned PennVet dataset significantly outperformed randomly permuted patient datasets for all three diseases (P < 0.05) for both precision and recall demonstrating that we were able to replicate known disease – breed associations in the literature. Our framework can be applied at other veterinary hospitals to produce large-scale veterinarian datasets for high-throughput discovery research.

We validated our data-cleaning framework by testing the cleaned PennVet dataset for known breed – disease associations found in the literature. Recall ranged from 31.6% to 83.3% depending on the disease of interest (Table 3) indicating that we were able to retrieve the expected disease – breed proclivities after applying our framework to PennVet data. Precision ranged from 60.0% to 83.3% (Table 3). The results for atrial fibrillation performed equally well in terms of both precision and recall – both at 83.3% (Table 3) because few breeds were associated in the literature (N = 6) and few results were returned using our algorithm (N = 6). In addition, atrial fibrillation is easier to diagnose require an electrocardiogram (ECG) alone and does not require as many diagnostics (e.g., biopsy to detect osteosarcoma)^42^.

Both Beagles and Golden Retrievers were reported to be at risk for mitral valve disease in the literature^22,43^. However, we failed to find these breeds at risk (Table 2**)** despite both being in the top 25 breeds treated at PennVet (Table 1). We also failed to find poodles associated with mitral valve disease. One study found that only toy and miniature Poodles were associated with mitral valve disease^25,26^ without reporting an association between standard Poodles and mitral valve disease. Another study found poodles in general (unclear what size of Poodle)^24^. Our dataset contains all three different sized poodles as distinct breeds, and we found none to be associated with mitral valve disease. Therefore, perhaps our distinction among the sizes of the poodles may have lowered our power to detect an association. None of the three sized Poodle breeds were in our top 25 dog breeds seen at PennVet (Table 1). Some of the studies reporting increased risk of mitral valve disease in Golden Retrievers appeared to be investigating congenital heart disease in general^43^. Congenital heart disease includes mitral valve dysplasia, which differs from the inherited degenerative mitral valve disease seen in small breed dogs. This mitral valve dysplasia was found in Golden Retrievers by the prior study^43^. However, there are some limitations with the Golden Retriever study, including its small sample size (13 Golden Retrievers), which may have biased their results^43^. Hence our findings may be closer to the truth, further research is needed to validate this. Bull Terriers also are known to acquire mitral valve dysplasia. We found Bull Terriers are increased risk for mitral valve disease in our study (Table 2), but not Golden Retrievers. The cleaned PennVet database could potentially include both mitral valve dysplasia (which is much rarer then degenerative mitral valve disease) and degenerative mitral valve disease, which could be affecting our breed-disease association results.

We compared our breed association results for mitral valve disease to several studies, including Mattin *et al*.^24^. In some cases the odds ratios for each breeds’ risk of developing mitral valve disease differed from our study. For example, the Cavalier King Charles Spaniel (CKCS) was reported as having an OR of 28.74 (95% CI: 20.41–40.48) in the Mattin *et al*. study versus our results showing an OR of 14.55 (95% CI: 11.44, 1834). This is likely due to a difference in the statistical methods used. Mattin *et al*. performed a univariate logistic regression analysis where each breed was compared against the ‘crossbred’ or mixed breed population. In our study, we compared each breed against the overall general population^24^. Therefore, our estimates are not as dependent on the mixed breed population. Studies that compare breeds against a ‘mixed’ breed are difficult to replicate at other sites due to different breed distributions among the ‘mixed’ breed group. For example, the Veterinary practices in England could have more Yorkshire Terrier mixes and in Philadelphia we may have more Labrador Retriever mixes. Because we do not know the breed makeup of our ‘mixed breeds’, we chose to assess mixed breeds separately and not to use them as the comparator group. This likely accounts for the differences in the reported ORs between the two studies.

We found that the smaller breed dogs were at increased risk of inherited forms of degenerative mitral valve disease confirming the literature that smaller dog breeds are predisposed to degenerative mitral valve disease^44^. We found several small breeds at increased risk of mitral valve disease including the Maltese (N = 1121) confirming the literature reports for that breed’s association (Table 2). We did not find the Beagle (N = 1143, Table 1) at risk for mitral valve disease in our dataset despite a high prevalence of the breed. However, another study using the VetCompass data did not find an association between beagles and mitral valve disease^24^, which could point to limitations in the study that did report a relationship^45^. Interestingly, we found that the Pug was protected against mitral valve disease (OR = 0.24, CI: 0.05, 0.70, Table 2) being the only small-breed dog protected against the disease in our study.

Interestingly, we found that the American Pit Bull Terrier was protected against mitral valve disease (Table 2). In the literature, the Staffordshire Bull Terrier^24^ was reported to be protected against mitral valve disease. These two are similar genetically yet distinct breeds. Interestingly, we did not find Staffordshire Bull Terriers to be protected against mitral valve disease at PennVet even though they are known to be protected^24^, but we did find that the American Pit Bull Terrier was protected against mitral valve disease. Therefore, our finding is novel, but not surprising given the known protection against mitral valve disease among Staffordshire Bull Terriers. Anecdotally, owners in the USA sometimes report their dogs as Staffordshire Bull Terriers when they are American Pit Bull Terriers given the negative connotation of the ‘Pit Bull’ breeds. However, the “Pit Bull Terrier” is also a banned breed in the United Kingdom (UK) while the Staffordshire Terrier is an approved breed^46^. Therefore, it is possible that we found the association in PennVet because these dogs are legal in the USA while illegal in UK. Hence, the VetCompass dataset would not have access to ‘American Pit Bull Terrier’ dogs due to their legal status in the UK.

For osteosarcoma, we achieved high precision among at-risk breeds (75.0%, p = 0.003) and lower precision if we include the breeds protected against osteosarcoma (60.0%, p = 0.036, Table 3). To our knowledge, no prior study has investigated breeds for protection against osteosarcoma. Therefore, our discovery that Yorkshire Terriers, and Shih Tzu dogs are protected against osteosarcoma may be a novel clinically important finding (Table 5). For this informatics-methods study, we are focused on replicating the known findings and therefore, the precision of 75.0% among the at-risk breeds is the most appropriate because at-risk breeds were reported in the literature. Therefore, adequate comparison would only involve investigating significantly at risk breeds (i.e., OR >  = 1).

Other replication issues could be due to certain breeds being rare at PennVet. A Swedish study^34^ reported several breeds at risk for osteosarcoma that are rarely seen at PennVet, including the Leonberger (N = 31 at PennVet), Hovawart (N = 0 at PennVet), and Flat-coated Retriever (N = 46 at PennVet). Interestingly, the Anatolian Shepherd Dog was found to be at risk for osteosarcoma at PennVet (Table 5). Only 14 Anatolian Shepherd dogs were seen at PennVet between 2000 and 2017, and 2 had osteosarcoma resulting in an OR = 45.90 (CI: 4.97, 208.26) (Table 5). To our knowledge, no prior study has indicated that Anatolian Shepherd Dogs are at increased risk of osteosarcoma. However, the Anatolian Shepherd is a rare breed and therefore prior studies may not have included the Anatolian Shepherd. PennVet has a well-known canine cancer center for treating osteosarcoma, which could result in enrichment, of certain dog breeds and diseases because owners travel to PennVet for treatment.

Another potentially novel finding is the association between bullmastiff and risk of osteosarcoma (OR = 10.90, CI: 4.60, 22.15, Table 5). A relationship between the Mastiff breed and risk of osteosarcoma has been described previously^22,35^ and we failed to replicate it here at PennVet (Table 5). However, bullmastiffs have not been reported to be at risk for osteosarcoma previously. Three commonly treated breeds at PennVet – Golden Retrievers, Boxers and German Shepherds with 2,987, 2,128 and 2,693 dogs respectively – were not associated with increased risk of osteosarcoma, which lowers the recall (31.6%).

Limitations of our study include being potentially underpowered for detecting associations among certain dog breeds that are not commonly treated at PennVet, which we reported in our recall statistic. For example, the small dog breed – American Pit Bull Terrier is very common at PennVet and is the third most common dog breed (N = 4241, Table 1). The American Kennel Club (AKC) published the most popular dog breeds in 2018^47^, which differ in frequency at PennVet vs. the AKC’s USA statistics. For example, at PennVet, Labrador Retrievers were 2^nd^ most common (vs. 1^st^ in USA), German Shepherds were 5^th^ most common (vs. 2^nd^ in USA), Golden Retrievers were 4^th^ most common (vs. 3^rd^ in USA), French Bulldogs were 24^th^ most common (vs. 4^th^ in USA) and Bulldogs were 10^th^ most common (vs. 5^th^ in USA). Overall certain dog breeds (e.g., American Pit Bull Terrier) were more common in Philadelphia versus the USA in general and other regions. Also our dog population are principally pets and not AKC registered dogs used in breeding as reported in our previous study^7^. Therefore, breed information may be imprecise because it is self-reported by owners. In addition, our ‘mixed canine’ dogs were not identified as belonging to the dominant breed in the mix, unless it belonged to a ‘breed’ reported by the owners, such as ‘Goldendoodle’ and ‘Labradoodle’. Therefore, the specific proportion of breeds in our ‘mixed canine’ population could be specific to mixed canines in Philadelphia. Further, our population contains a very small proportion of intact (i.e., not castrated, spayed or neutered) dogs, <0.5% (419/84405). Therefore if some disease – breed associations reported in the literature vary based on whether the animal is spayed/neutered/castrated vs. intact, then we would not be able to replicate results of associations with intact animals. For mitral valve disease, while we were able to distinguish between congenital and acquired mitral valve disease (we report on acquired mitral valve disease), we were unable to distinguish between the cause of acquired mitral valve disease (e.g., mitral valve disease due to valve degeneration versus dilated cardiomyopathy) and therefore some differences between our study and those in the literature that distinguish these two types of mitral valve disease is expected.

Another limitation of our study is that we utilized a secondary source to identify primary sources that indicate that a dog breed was at risk for certain diseases^22^. We used this secondary source to easily identify studies that support the relationship between specific dog breeds and the three diseases in this study. However, not all studies are created equal and there are limitations with some of the studies. Rather than carefully review every study for statistical flaws and other issues, we chose to trust our reference as a guide. We then validated the findings from PennVet against this gold-standard. However, some studies may not adequately capture the true relationship between a particular dog breed and a disease (e.g., being under powered, using the wrong statistical test, and so forth). We recognize this as a limitation of our study. However, we are confident in our method given our consistent ability to significantly outperform random with regards to replicating the literature (Fig. 5, Table 3).

## Conclusion

Our study demonstrates that a clinical data repository can be constructed using canine data obtained during routine clinical care at a veterinary hospital (PennVet). Our framework consists of two phases: 1) a semantic data-cleaning phase followed by 2) a domain-specific data cleaning phase. This two-step process allowed us to maximize data retention (99.8% of data retained), while ensuring that the data included in the repository was sufficient quality for research. We assessed the data quality by comparing the results from PennVet for disease – breed associations with those found in the literature. The precision ranged from 60.0% to 83.3% while the recall ranged from 31.6% to 83.3%. Note that novel findings reported by our algorithm would lower precision as precision is based on what is known. Our validated framework could be used at other veterinary hospitals to produce large-scale datasets of disease-related information from companion animals that would be useful for high-throughput discovery research.

## Supplementary information

Dataset 1

Dataset 2

## References

[1] Boland MR, Kashyap A, Xiong J, Holmes J, Lorch S (2018). Development and validation of the PEPPER framework (Prenatal Exposure PubMed ParsER) with applications to food additives. Journal of the American Medical Informatics Association.

[2] Gurda BL, Bradbury AM, Vite CH (2017). Focus: Comparative Medicine: Canine and Feline Models of Human Genetic Diseases and Their Contributions to Advancing Clinical Therapies. The Yale journal of biology and medicine.

[3] Casal M. L. & Me, H. In *Mucopolysaccharidoses Update (Metabolic Diseases - Laboratory and Clinical Research)* Ch. 35, 697–712 (2019).

[4] Boland, M. R., Dziuk, E., Kraus, M. & Gelzer, A. Cardiovascular Disease Risk Varies by Birth Month in Canines. *Scientific Reports* **8**, 10.1038/s41598-41018-25199-w (2018). 10.1038/s41598-018-25199-w PMC5958072 29773810

[5] Karlsson EK (2013). Genome-wide analyses implicate 33 loci in heritable dog osteosarcoma, including regulatory variants near CDKN2A/B. Genome biology.

[6] Withrow, S. J., Powers, B. E., Straw, R. C. & Wilkins, R. M. Comparative aspects of osteosarcoma. Dog versus man. *Clinical orthopaedics and related research*, 159–168 (1991). 1884536

[7] Boland MR (2018). Uncovering exposures responsible for birth season – disease effects: a global study. Journal of the American Medical Informatics Association.

[8] Boland MR, Shahn Z, Madigan D, Hripcsak G, Tatonetti NP (2015). Birth month affects lifetime disease risk: a phenome-wide method. Journal of the American Medical Informatics Association.

[9] Li, L., Boland, M. R., Miotto, R., Tatonetti, N. P. & Dudley, J. T. Replicating Cardiovascular Condition-Birth Month Associations. *Scientific Reports* **6**, 33166, 10.1038/srep33166, https://www.nature.com/articles/srep33166-supplementary-information (2016). 10.1038/srep33166 PMC5021975 27624541

[10] Overhage JM, Ryan PB, Reich CG, Hartzema AG, Stang PE (2011). Validation of a common data model for active safety surveillance research. Journal of the American Medical Informatics Association.

[11] Safran C (2007). Toward a national framework for the secondary use of health data: an American Medical Informatics Association White Paper. Journal of the American Medical Informatics Association.

[12] Von Eschenbach AC, Buetow K (2006). Cancer informatics vision: caBIG™. Cancer informatics.

[13] Cruz-Correia RJ (2009). Data quality and integration issues in electronic health records. Information discovery on electronic health records.

[14] Smith-Akin KA, Bearden CF, Pittenger ST, Bernstam EV (2007). Toward a veterinary informatics research agenda: an analysis of the PubMed-indexed literature. International Journal of Medical Informatics.

[15] Smith RD, Williams M (2000). Applications of informatics in veterinary medicine. Bulletin of the Medical Library Association.

[16] Cheng, K., Baldwin, T. & Verspoor, K. In *Proceedings of the Australasian Language Technology Association Workshop* 70–78 (2017).

[17] Küker S (2018). The value of necropsy reports for animal health surveillance. BMC veterinary research.

[18] Hur, B., Hardefeldt, L., Verspoor, K., Baldwin, T. & Gilkerson, J. Using natural language processing and VetCompass to understand antimicrobial usage patterns in Australia. *Australian veterinary journal* (2019). 10.1111/avj.12836 31209869

[19] Boland MR, Karczewski KJ, Tatonetti NP (2017). Ten Simple Rules to Enable Multi-site Collaborations through Data Sharing. PLOS Computational Biology.

[20] Weiskopf NG, Weng C (2013). Methods and dimensions of electronic health record data quality assessment: enabling reuse for clinical research. Journal of the American Medical Informatics Association.

[21] Schubart JR, Einbinder JS (2000). Evaluation of a data warehouse in an academic health sciences center. International Journal of Medical Informatics.

[22] Gough, A., Thomas, A. & O’Neill, D. *Breed predispositions to disease in dogs and cats*. (John Wiley & Sons, 2018).

[23] Chetboul V (2004). Epidemiological, clinical, echo-doppler characteristics of mitral valve endocardiosis in Cavalier King Charles in France: a retrospective study of 451 cases (1995 to 2003). The Canadian Veterinary Journal.

[24] Mattin MJ (2015). Prevalence of and Risk Factors for Degenerative Mitral Valve Disease in Dogs Attending Primary-care Veterinary Practices in England. Journal of Veterinary Internal Medicine.

[25] Buchanan, J. Prevalence of cardiovascular disorders. *Textbook of canine and feline cardiology: principles and clinical practice*, 457–470 (1999).

[26] Oyama M, Levy R (2010). Insights into serotonin signaling mechanisms associated with canine degenerative mitral valve disease. Journal of veterinary internal medicine.

[27] Tidholm A, Jonsson L (1997). A retrospective study of canine dilated cardiomyopathy (189 cases). Journal of the American Animal Hospital Association.

[28] Menaut P, Bélanger MC, Beauchamp G, Ponzio NM, Moïse NS (2005). Atrial fibrillation in dogs with and without structural or functional cardiac disease: a retrospective study of 109 cases. Journal of Veterinary Cardiology.

[29] Tidholm A, Jonsson L (1996). Dilated cardiomyopathy in the Newfoundland: a study of 37 cases (1983–1994). Journal of the American Animal Hospital Association.

[30] Meurs KM, Miller MW, Wright NA (2001). Clinical features of dilated cardiomyopathy in Great Danes and results of a pedigree analysis: 17 cases (1990–2000). Journal of the American Veterinary Medical Association.

[31] Vollmar AC (2000). The prevalence of cardiomyopathy in the Irish wolfhound: a clinical study of 500 dogs. Journal of the American Animal Hospital Association.

[32] Saunders, A., Gordon, S. & Miller, M. Canine atrial fibrillation. *Compendium Continuing Education for Veterinarians* **31** (2009). 20180218

[33] Rosenberger JA, Pablo NV, Crawford PC (2007). Prevalence of and intrinsic risk factors for appendicular osteosarcoma in dogs: 179 cases (1996–2005). Journal of the American Veterinary Medical Association.

[34] Egenvall A, Nødtvedt A, von Euler H (2007). Bone tumors in a population of 400 000 insured Swedish dogs up to 10 y of age: incidence and survival. Canadian Journal of Veterinary Research.

[35] Ru G, Terracini B, Glickman L (1998). Host related risk factors for canine osteosarcoma. The Veterinary Journal.

[36] Mueller F (2005). Palliative radiotherapy with electrons of appendicular osteosarcoma in 54 dogs. In vivo.

[37] Ramirez O (1999). Palliative radiotherapy of appendicular osteosarcoma in 95 dogs. Veterinary Radiology & Ultrasound.

[38] Mauldin GN, Matus RE, Withrow SJ, Patnaik AK (1988). Canine osteosarcoma: treatment by amputation versus amputation and adjuvant chemotherapy using doxorubicin and cisplatin. Journal of Veterinary Internal Medicine.

[39] Priester, W. A. & McKay, F. W. The occurrence of tumors in domestic animals. *National Cancer Institute Monograph*, 1–210 (1980). 7254313

[40] Kistler, K. Canine osteosarcoma: 1462 cases reviewed to uncover patterns of height, weight, breed, sex, age and site involvement. *Phi Zeta Awards, University of Pennsylvania, School of Veterinary Medicine* **198** (1981).

[41] Goldschmidt, M. H. & Thrall, D. E. Malignant bone tumors in the dog. *NEWTON CD; NUNAMAKER, DM Textbook of Small Animal Orthopaedics. Ithaca: International Veterinary Information Service* (1985).

[42] Dilaveris PE (1998). Simple electrocardiographic markers for the prediction of paroxysmal idiopathic atrial fibrillation. American heart journal.

[43] Tidholm A (1997). Retrospective study of congenital heart defects in 151 dogs. Journal of Small Animal Practice.

[44] Atkins C (2009). Guidelines for the diagnosis and treatment of canine chronic valvular heart disease. Journal of veterinary internal medicine.

[45] Vörös K (2015). Occurrence of mitral valve insufficiency in clinically healthy Beagle dogs. Acta Veterinaria Hungarica.

[46] UK. Controlling your dog in public: Banned Dogs. https://www.gov.uk/control-dog-public/banned-dogs, Accessed in October 2019 (2019).

[47] AKC. Most Popular Breeds, https://www.akc.org/most-popular-breeds, Accessed in October, 2019 (2019).

